# Effects of Divided Attention at Retrieval on Conceptual Implicit Memory

**DOI:** 10.3389/fpsyg.2016.00005

**Published:** 2016-01-21

**Authors:** Matthew W. Prull, Courtney Lawless, Helen M. Marshall, Annabella T. K. Sherman

**Affiliations:** Department of Psychology, Whitman CollegeWalla Walla, WA, USA

**Keywords:** divided attention, implicit memory, retrieval, repetition priming, memory, long-term, attention

## Abstract

This study investigated whether conceptual implicit memory is sensitive to process-specific interference at the time of retrieval. Participants performed the implicit memory test of category exemplar generation (CEG; Experiments 1 and 3), or the matched explicit memory test of category-cued recall (Experiment 2), both of which are conceptually driven memory tasks, under one of two divided attention (DA) conditions in which participants simultaneously performed a distracting task. The distracting task was either syllable judgments (dissimilar processes), or semantic judgments (similar processes) on unrelated words. Compared to full attention (FA) in which no distracting task was performed, DA had no effect on CEG priming overall, but reduced category-cued recall similarly regardless of distractor task. Analyses of distractor task performance also revealed differences between implicit and explicit memory retrieval. The evidence suggests that, whereas explicit memory retrieval requires attentional resources and is disrupted by semantic and phonological distracting tasks, conceptual implicit memory is automatic and unaffected even when distractor and memory tasks involve similar processes.

## Introduction

When we retrieve information from memory, we often do so in the context of an ongoing activity. For example, we may try to remember the melody or lyrics to a song while we are driving, or we may try to recall a person’s name while having a conversation with them. Under such situations, our attention is said to be divided between memory retrieval and the demands of another task. How is memory affected under such conditions compared to situations in which an ongoing task is absent and one can devote full attention (FA) to retrieval? Are different types of memory affected differently when attention is divided at the time of retrieval?

Somewhat surprisingly, relatively few studies have tackled these questions. Most studies of divided attention (DA) and memory have focused on encoding rather than retrieval, and those studies show clearly that DA affects many kinds of memory. Specifically, using a procedure in which participants perform a distracting or secondary task simultaneously while encoding new information, researchers have shown that performance on explicit memory tests of recall or recognition is sharply reduced compared to a FA condition in which attention is not diverted at encoding. In these studies, virtually any distracting task has a negative effect on memory (Murdock, 1965; Baddeley et al., 1984; Fisk and Schneider, 1984; Craik et al., 1996; Anderson et al., 1998; Naveh-Benjamin et al., 1998, 2000; see Mulligan, 2008, for a review).

Attention at encoding is also necessary for implicit memory, in which memories are revealed in performance changes on tasks that make no reference to study events and do not require the deliberate retrieval processes that are involved in explicit memory. In such studies, encoding-phase DA reduces repetition priming, a form of implicit memory, on some but not all implicit memory tests (e.g., Parkin et al., 1990; Schmitter-Edgecombe, 1996; Szymanski and MacLeod, 1996; Mulligan, 1998; Gabrieli et al., 1999; Light et al., 2000; Rajaram et al., 2001; Mulligan and Peterson, 2008; see Spataro et al., 2011 for review). Two theoretical perspectives help understand the variation in outcomes. The *transfer-appropriate processing perspective*, or TAP, divides implicit memory tests into perceptually driven and conceptually driven categories. Perceptually driven tests such as word stem completion (complete *squ___* with the first word that comes to mind) and lexical decision (is *squash* a word?) present stimuli in degraded, speeded, or other perceptually challenging forms. *Perceptual priming* occurs when studied items come to mind, or are processed faster, compared to unstudied items. Such priming is said to be perceptual in nature because it is affected by changing the perceptual similarity between test stimuli and study stimuli. In contrast, conceptually driven tests, such as word association (*gourd* - ?) or category verification (is *squash* a vegetable?), present test stimuli to which participants respond based on semantic knowledge. *Conceptual priming* occurs when studied items come to mind, or are processed faster, compared to unstudied items, but in this case priming is said to be conceptual because it is sensitive to study-phase manipulations of conceptual processing, not manipulations of study-to-test perceptual similarity (Roediger and McDermott, 1993). DA at encoding affects priming on many conceptually driven implicit memory tests and does not affect many perceptually driven tests, although there are some exceptions to that pattern (e.g., Light et al., 2000; Mulligan and Hornstein, 2000). That pattern is explained by the TAP perspective by assuming that encoding-phase DA hampers conceptual but not perceptual processing of stimuli, thereby affecting conceptual but not perceptual priming (Roediger and McDermott, 1993).

Alternatively, the *identification-production perspective* divides implicit memory tests into those that require identification of a stimulus cue or classification of one of its attributes (e.g., lexical decision and category verification tests, above), and those that cannot be completed by merely identifying test cues but instead require participants to go beyond the given cue information and produce a response from one of several candidate options (e.g., word stem completion and word association tests above; Gabrieli et al., 1994, 1999). Identification and production priming tasks can be either perceptual or conceptual in nature, and the identification-production perspective states explicitly that attention plays a critical role in production priming but far less so in identification priming. Attention at encoding may assist with resolving competition among multiple candidate responses at the time of test; this resolution of competition is thought to be common in production priming but not identification priming tasks (Gabrieli et al., 1999). Consistent with these views, encoding-phase DA impacts many production priming tasks without affecting many identification priming tasks, although as with TAP, there are a few exceptions to this pattern (e.g., Light and Prull, 1995; Schmitter-Edgecombe, 1999).

Less is known how explicit and implicit memory are affected when encoding takes place under FA conditions and DA is manipulated at retrieval. With respect to explicit memory, DA at retrieval was initially found to have little or no effect on recall and recognition compared to FA at retrieval, a surprising result that led to the view that explicit memory retrieval was relatively automatic and did not require attentional resources (the *automatic retrieval hypothesis*; Baddeley et al., 1984). Nevertheless, DA at retrieval is associated with robust performance decrements on the distracting task when compared to performance in a baseline condition in which the distracting task is performed alone, in the absence of the memory task (Craik et al., 1996; Naveh-Benjamin et al., 1998). This finding led Craik et al. (1996) to conclude that retrieval is not automatic in the strictest sense, as it clearly requires attentional resources, but that retrieval is protected or privileged over other ongoing tasks and is therefore affected little, if at all, by distraction.

Nevertheless, under some conditions, retrieval-phase DA effects on explicit memory can be substantial (Jacoby, 1991; Hicks and Marsh, 2000; Lozito and Mulligan, 2006). One crucial factor appears to be whether the distracting task and the memory task use similar materials. In a series of studies, Fernandes and Moscovitch (2000, 2002, 2003; Skinner and Fernandes, 2008) found that distracting tasks whose materials mismatched (e.g., digits) the to-be-remembered material (e.g., words) had little impact on recall performance, but distracting tasks whose materials matched the to-be-remembered material (i.e., both memory and distracting tasks used word stimuli) had considerable negative effects on recall. This *material-specific DA effect* suggests that explicit memory retrieval is most affected when other concurrent tasks compete for information in a common representational system (Fernandes and Moscovitch, 2000).

Very little is known how DA affects implicit memory at the time of retrieval, and only a handful of studies have systematically investigated this issue (Clarke and Butler, 2008; Lozito and Mulligan, 2010). These studies have generally provided good support for the automatic retrieval hypothesis as applied to implicit memory. Clarke and Butler (2008) found that a syllable monitoring distracting task had no effect on word-stem completion priming, in which words are studied and participants respond to word stems (e.g., *sta___*) with the first word that comes to mind. However, the same distracting task had a significant negative effect on the matched explicit test of cued recall.^1^ Lozito and Mulligan (2010) used a variety of distracting tasks and multiple implicit memory tasks such as perceptual identification, word-stem completion, and category exemplar generation (CEG) to investigate retrieval-phase DA effects on implicit memory. Priming was unaffected in every case, regardless of whether distracting tasks required frequent or occasional responses (which can sometimes determine whether encoding-phase DA effects occur for implicit memory; see Mulligan, 2003; Mulligan et al., 2007), and regardless of whether distracting task materials overlapped with the implicit memory tasks. Together, these studies provide good support for the automaticity hypothesis, and they join additional studies that have reported little or no effect of retrieval-phase DA on other automatic uses of memory such as artificial grammar learning, false fame, or estimates of familiarity in recognition (Jacoby et al., 1989; Jacoby, 1991; Helman and Berry, 2003).

The purpose of this study is to test the automatic retrieval hypothesis further by manipulating the overlap in processes, rather than materials, shared by the distracting task and implicit memory task. Fernandes and Guild (2009) have argued that *process-specific DA effects* can occur in explicit memory, in which memory performance diminishes when both memory and distractor tasks compete for the same set of processes at the time of retrieval. When tasks compete for different processes, interference is not as great and memory performance is affected less. In support of these assertions, Fernandes and Guild (2009) found that, relative to an FA condition, recognition memory for spatial patterns was reduced more by a distracting task involving visuospatial judgments about letters that were presented aurally (deciding whether letters have curved lines when printed), than by a distracting task that involved making phonological judgments on the same letters (deciding whether each letter rhymed with “e”). The opposite pattern of results was found when the memory task involved words: recognition performance diminished more under the phonological distractor task than under the visuospatial distractor task. Note that this outcome cannot be due to a material-specific DA effect, as the materials in the distractor task remained constant. In another study (Wammes and Fernandes, 2015), participants were given recognition tests for upright or inverted faces that they had studied. Memory for upright faces is thought to depend on processing holistic or configural interrelationships between face details, whereas memory for inverted faces is thought to depend on processing specific, stand-alone features. Accordingly, recognition memory for upright faces was negatively affected more by a distracting task that also required holistic processing than one that required featural processing, whereas the reverse outcome occurred when participants studied and recognized inverted faces. Other such demonstrations of process-specific interference have been reported in a variety of short-term and working memory tasks (e.g., Brooks, 1968; den Heyer and Barrett, 1971; Pellegrino et al., 1975; Logie et al., 1990; Robbins et al., 1996).

The current study examined implicit memory under DA conditions at the time of retrieval, using distractor tasks that either did or did not overlap with the implicit memory task in terms of processes. We used the CEG task in this investigation because it should be particularly sensitive to DA effects according to the TAP and identification-production frameworks. The CEG task requires participants to provide the first example of a category (e.g., *crow*) in response to a category name (e.g., *a type of bird*). Priming is revealed to the extent that studied exemplars are produced more often than the baseline rate of responding when exemplars are unstudied. The CEG task is classified as a conceptually driven implicit memory test and involves stimulus production, and both TAP and the identification-production accounts converge in predicting DA effects on priming on such a task. Indeed, encoding-phase DA produces robust decrements in CEG priming (see Spataro et al., 2011).

Lozito and Mulligan (2010) found no retrieval-phase DA effect on CEG priming, but they manipulated only the similarity of materials shared by the distracting task and memory task. We address the open question of whether such priming is sensitive to process-specific interference. In the present study, we used one distractor task that required phonological processing of words (syllable judgments). Phonological processing overlaps relatively little with the semantic or conceptual processes involved in generating an example to a category. We also used a distractor task that required semantic processing of words (abstract/concrete judgments), which overlaps considerably more with the conceptual processes underlying the CEG task. From a neuroscience perspective, these same phonological and semantic judgment tasks activate different regions within the left prefrontal lobe (e.g., Poldrack et al., 1999). The fact that phonological distractor tasks did not affect conceptual priming (Lozito and Mulligan, 2010) could reflect a successful coordination of different processes across different brain regions. In contrast, semantic judgments to words and conceptual priming involve activations and deactivations within the same left prefrontal region (e.g., Raichle et al., 1994; Gabrieli et al., 1996). Thus, a distractor task that incorporates semantic processes should be most likely to affect priming in the CEG task.

## Experiment 1

In Experiment 1, participants studied a list of category exemplars under FA. They then completed a CEG task under conditions of FA, DA involving syllable judgments on distractor words, or DA involving semantic judgments on distractor words. In all cases, the material overlap between distractor task and memory task was held constant, only the type of processing performed on the distractor stimuli varied. According to the automatic retrieval hypothesis, priming on the CEG task should be unaffected by either of the two DA tasks. However, if priming is sensitive to process-specific interference, the semantic distractor task should reduce priming relative to the other two conditions.

The automatic retrieval hypothesis can also be evaluated by examining how the CEG task influences distractor task performance, which we call *distractor task costs*. We followed Lozito and Mulligan’s (2010) method for examining distractor task costs. First, *global costs* can be assessed by comparing performance on the distractor task when performed under DA (i.e., when the CEG task is performed simultaneously) to that of a baseline condition in which the distractor task is performed alone. Worse performance on the distractor task under DA conditions, compared to baseline, would indicate that the CEG task itself is attention-demanding. However, such information would not speak to the attentional demands of implicit memory retrieval. *Specific costs* do so, however. Such costs refer to performance decrements on the distractor task on trials where category names correspond to studied exemplars (*studied trials*), compared to category names that correspond to unstudied exemplars (*unstudied trials*) in the DA conditions. Such costs, either to distractor task accuracy or response time (RT), would signal that implicit memory retrieval is attention-demanding. However, if the automatic retrieval hypothesis is correct, distractor task performance should not be negatively affected in studied trials (i.e., no specific costs). Lozito and Mulligan (2010) reported precisely that outcome, and in fact found specific benefits in some cases such that studied trials facilitated the distractor task by increasing accuracy or decreasing reaction times. In the present experiment, we included a baseline phase of distractor task trials so that we could calculate global attentional costs, and we analyzed distractor performance separately for studied and unstudied trials in the DA conditions in order to assess specific costs.

### Method

#### Participants

Forty-eight people (31 women, 17 men) participated in Experiment 1 for course credit or for pay. In this and in all subsequent experiments, participants were undergraduate students of traditional age (18–22 years) enrolled at Whitman College. Written informed consent was obtained from all participants. All experiments reported in this article were carried out with approval from the Institutional Review Board at Whitman College.

#### Materials and Design

We selected one exemplar from each of 60 different categories to create a master list of 60 critical items. The items and categories were selected from several different norms and the items were never among the top three responses for their respective category (Hunt and Hodge, 1971; McEvoy and Nelson, 1982; Van Overschelde et al., 2004; Yoon et al., 2004). We avoided proper names and compounds (e.g., *flip-flop, foxtrot*). This master list was divided into two lists, each 30 items in length. We placed two primacy buffers at the beginning and two recency buffers at the end of each list to create two 34-item lists, one of which served as the studied, or old, list while the other served as the unstudied, or new, list. The presentation of these two lists as studied or unstudied was counterbalanced across participants.

The test included 90 category names. Thirty category names corresponded to the list of 30 studied exemplars that was presented during the study phase, 30 category names corresponded to the remaining exemplars on the second list, which were unstudied, and 30 filler category names corresponded to none of the exemplars but were included in the test in an effort to disguise the connection between the study phase and test phase.

The test phase occurred under conditions of FA, DA involving syllable judgments (DA-Syllable), or DA involving semantic judgments (DA-Semantic). In all cases during the test, participants heard 180 nouns spoken through the computer speakers and saw the 90 category names on the computer screen. None of the 180 words was a reasonable exemplar of any category tested, and these words remained constant for all participants. A quarter of these words had two syllables and referred to abstract entities (*reason, beauty*), another quarter were two-syllable words that referred to concrete entities (*cashew, lever*), a third quarter were not two-syllable words and referred to abstract entities (*joy, creation*), and the last quarter were not two-syllable words and referred to concrete entities (*acrobat, foam*). Participants in the FA condition ignored the spoken words and focused all their attention on performing the CEG task. Participants in the two DA conditions responded to the spoken words with either syllable judgments or semantic judgments while simultaneously completing the CEG task.

The baseline task consisted of a separate group of 120 nouns with similar properties as the DA distractor words just described. The baseline words also remained constant across participants and were divided into quarters according to two/not-two syllable and abstract/concrete categories.

#### Procedure

Participants were randomly assigned to FA, DA-Syllable, or DA-Semantic conditions and were tested individually. All participants began with the study phase in which one of the two lists of exemplars was presented visually. Participants made pleasantness judgments for each exemplar using keypress responses (1 = u*npleasant*, 2 = *neutral*, 3 = *pleasant*). Each word was presented individually for 3 s with a 500 ms blank screen interstimulus interval (ISI). Encoding was incidental, as participants were not told that their memory for the words would be tested later, or that the words were exemplars of various taxonomic categories. The study phase was followed by a choice reaction time task administered on the computer that lasted about 2 min and served merely as a filler task in order to obscure the nature of the experiment.

To obtain baseline rates of performance on the syllable judgment task (for DA-Syllable participants) or the semantic judgment task (for DA-Semantic participants), we presented the 120-word list aurally for participants to make judgments appropriate to the condition to which they were assigned. Each word was spoken by a male voice one at a time and followed by silence so that 3 s elapsed between the onset of each spoken word. Sixty of these words were presented after the choice RT task, immediately before the CEG task that is described in the next paragraph. The remaining 60 words were presented immediately after the CEG task. Participants therefore heard 60 words in each of two rounds of baseline performance. For those in the DA-Syllable condition, participants pressed the slash key (/) on the keyboard, labeled “two,” if the word contained two syllables, or they pressed the z key, labeled “not two,” if the word contained anything other than two syllables. For those in the DA-Semantic condition, participants pressed the slash key (/), labeled “concrete,” if the word represented a concrete entity, or they pressed the z key, labeled “abstract,” if the word represented an abstract entity. Participants in the FA condition completed only the first round of the baseline task, and for them the task served merely as a second distractor task before they advanced to the CEG task. Half of the FA participants completed one round of syllable judgments and the other half completed one round of semantic judgments. In all cases, no more than three two-syllable words, three not-two syllable words, three abstract, or three concrete words occurred consecutively.

**FIGURE 1:**
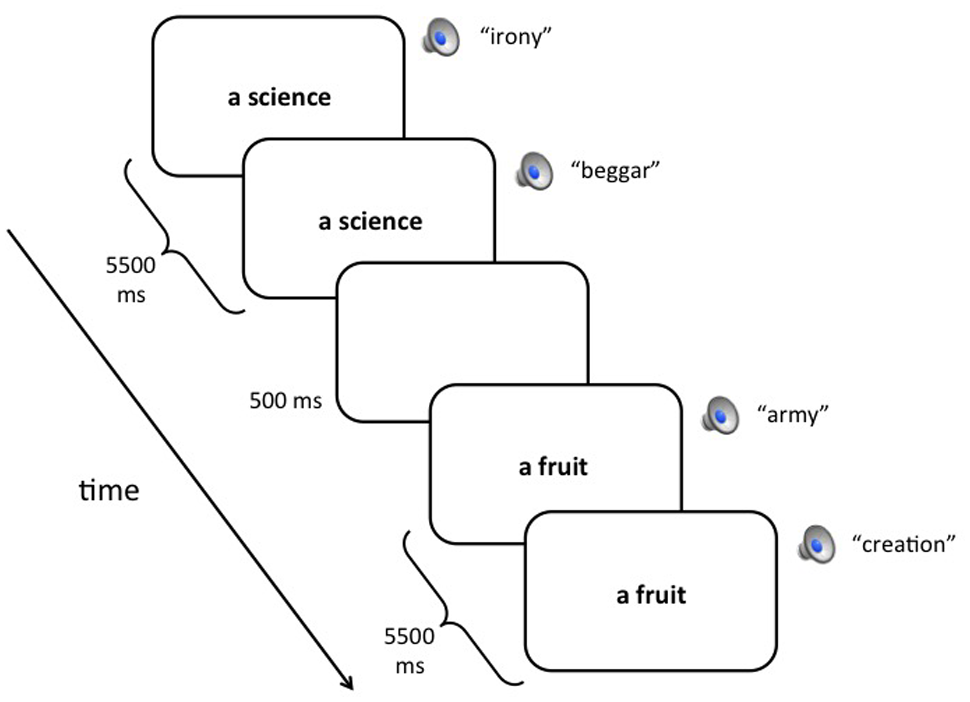
**Schematic of the category exemplar generation test**. At the beginning of each trial, a category name appeared and remained continuously on the screen for 5.5 s. A spoken word was also presented simultaneously with the onset of the category name (simultaneous distractor). Three seconds later, a second spoken word was presented (delayed distractor). Each trial was separated by a 500 ms blank screen. In the full attention (FA) condition, participants responded to the category names with the first example that came to mind. In the divided attention (DA) conditions, participants additionally responded to the spoken words, either by making syllable judgments (DA-Syllable) or abstract/concrete judgments (DA-Semantic).

For the CEG task, participants saw 90 category names and heard 180 words (see **Figure 1** for a schematic). Category names (e.g., “a dance”) were presented one at a time and remained on the screen for 5.5 s followed by a 500 ms blank screen ISI. Except for the first five category names, which were all fillers, no more than two items from any of the three categories (studied, unstudied, or filler) occurred consecutively. Two spoken words were heard for every one category name. One spoken word was presented simultaneously with the visual onset of the category name and a second spoken word was presented 3 s later, while the category name was still visible. As with the baseline trials, no more than three two-syllable words, three not-two syllable words, three abstract, or three concrete words occurred consecutively. All participants were instructed to use each category name as a cue to say aloud the first example of the category that came to mind. Those in the DA-Syllable and DA-Semantic conditions additionally responded to the spoken words, making two/not-two or abstract/concrete responses by keypress in the same way as during the first round of baseline trials. All DA participants were instructed to emphasize both tasks equally and to perform both tasks to the best of their abilities. Those in the FA condition were told to ignore the spoken words entirely, as they would only distract them from performing the CEG task, and to focus all their attention on completing the CEG task. Two short breaks were provided during the test, one after the 30th category name then another after the 60th category name.

After the CEG task, those in the DA-Syllable and DA-Semantic tasks completed the second round of baseline trials on the remaining 60 baseline words, and then completed an awareness questionnaire. Those in the FA condition proceeded immediately to the awareness questionnaire upon completion of the CEG task. The awareness questionnaire assessed the degree to which participants became aware of the connection between the study phase and the test phase. The awareness questionnaire was modeled after those used by others (Bowers and Schacter, 1990; Barnhardt and Geraci, 2008) and consisted of six funnel questions ranging from general (*what do you think was the purpose of the task in which you gave examples of various categories while hearing words?*) to specific (*if you noticed that some of the examples you gave were the words that you had rated for pleasantness, did you intentionally try to use those words as responses to the categories?*). Such questionnaires are routinely used in implicit memory research and provide valid methods for assessing awareness (Barnhardt and Geraci, 2008).

### Results

#### Encoding

Participants provided pleasantness ratings to 99.0, 99.6, and 99.8 percent of the critical words in the FA, DA-Syllable, and DA-Semantic conditions, respectively. These differences were not reliable by a one-way analysis of variance (ANOVA), *F*(2,45) = 1.50, *p* = 0.23 (unless stated otherwise, two-tailed α = 0.05 throughout).

#### Implicit Memory

The proportions of critical items produced on the CEG test for all participants are shown in the top half of **Table 1**. A one-way ANOVA on baseline rates of responding with critical words when they were unstudied revealed no reliable difference across groups, *F* < 1. Therefore, we computed priming scores as the proportion of studied critical items given at test minus the unstudied response rates. As can be seen in **Table 1**, priming was not reduced by DA, and we confirmed this impression with a one-way ANOVA conducted on the priming scores, *F*(2,45) = 1.32, *MSE* = 0.006, *p* = 0.28. Priming was reliably greater than zero when each condition was analyzed separately, all *t*(15)s ≥ 3.13, all *p*s < 0.01.

**Table 1:** Experiment 1 proportions of critical items produced on the implicit memory test of category exemplar generation (±1 SE in parentheses).

Condition	Studied items	Unstudied items	Priming
**Full sample**			
FA	0.14 (0.02)	0.08 (0.01)	0.07 (0.02)
DA-Syllable	0.17 (0.02)	0.06 (0.01)	0.11 (0.01)
DA-Semantic	0.13 (0.02)	0.06 (0.01)	0.07 (0.02)
**Unaware only**			
FA	0.16 (0.02)	0.06 (0.02)	0.10 (0.02)
DA-Syllable	0.14 (0.02)	0.05 (0.02)	0.09 (0.01)
DA-Semantic	0.10 (0.02)	0.06 (0.01)	0.04 (0.02)

*FA, full attention; DA, divided attention; Unaware Only, participants who did not report awareness of the relationship between test cues and studied words*.

We used responses to the questionnaire to classify participants as unaware of the relationship between study and test phases (*test-unaware*), aware of the relationship but did not use intentional retrieval strategies at test (*test-aware*), or aware of the relationship and used intentional retrieval strategies at test (*intentional*). When analyses were restricted to those who were test-unaware (*n* = 6, 8, and 11 in the FA, DA-Syllable, and DA-Semantic conditions, respectively), a reliable difference in priming emerged between the groups, *F*(2,22) = 4.42, *MSE* = 0.002, *p* = 0.02, $\eta_{p}^{2}$ = 0.29. As can be seen in the lower half of **Table 1**, priming in the DA-Semantic condition was reliably lower relative to the FA condition (*p* < 0.05) and nearly reliably lower relative to the DA-Syllable condition (*p* = 0.06). The DA-Syllable and FA conditions did not differ reliably from each other (*p* = 0.95). Thus, among test-unaware participants, the semantic DA task reduced priming relative to FA and DA-Syllable conditions.

#### Distracting Task

As described earlier, there are two ways in which distracting task performance can be analyzed. Global costs refer to the performance decrements on the distracting task when carried out under DA compared to baseline. Specific costs refer to the decrements on DA distractor task performance on trials where categories correspond to studied exemplars (studied trials) are compared to trials where categories correspond to unstudied exemplars (unstudied trials). The outcome patterns described next were the same regardless of whether we analyzed the full sample or only those that were classified as test-unaware, therefore only the results from the full sample are reported.

To assess global costs, we first calculated mean accuracy for the distractor task performed alone, averaged across both blocks of baseline trials, and compared performance to the same task when it was undertaken during DA in which the CEG task was also performed. Filler trials from the DA phase were excluded from these calculations, leaving 120 DA distractor task trials for analysis. Inspection of the left two columns of **Table 2** suggests that global costs occurred on the distracting task. Relative to baseline, accuracy decreased for the syllable and semantic tasks when either task was performed under DA. This impression was confirmed by a 2 (task: syllable, semantic) × 2 (block: baseline, DA) mixed ANOVA on accuracy that returned a main effect of block, *F*(1,30) = 134.99, *MSE* = 0.005, *p* < 0.001, $\eta_{p}^{2}$ = 0.82, as well as a main effect of task in which accuracy was reduced more for the semantic task than for the syllable judgment task, *F*(1,30) = 16.29, *MSE* = 0.009, *p* < 0.001, $\eta_{p}^{2}$ = 0.35. The interaction was non-reliable, *F* < 1.

**Table 2:** Experiment 1 accuracy and reaction time (RT) to distracting tasks.

	Global costs		Specific costs			
			DA-Studied		DA-Unstudied	
	Baseline	DA-Overall	Sim	Del	Sim	Del
Accuracy						
DA-Syllable	0.95	0.76	0.74	0.76	0.74	0.80
DA-Semantic	0.87	0.65	0.60	0.71	0.58	0.72
RT						
DA-Syllable	1061	1594	1865	1455	1777	1439
DA-Semantic	1256	1744	1940	1648	2037	1478

*DA, divided attention; Sim, simultaneous distractors; Del, delayed distractors*.

**Table 2** also suggests that global costs occurred for RTs on distractor trials, such that RTs increased on the distracting tasks when performed under DA. We first computed median RTs for each participant from correct responses to distractor task trials (excluding all fillers), and **Table 2** displays the mean median values for each condition across participants. A similar ANOVA on RTs returned a main effect of block, indicating slower RTs for DA than for baseline overall, *F*(1,30) = 182.24, *MSE* = 22,869, *p* < 0.001, $\eta_{p}^{2}$ = 0.86. There was also a main effect of task, indicating slower RTs for the semantic task than for the syllable judgment task overall, *F*(1,30) = 8.61, *MSE* = 55,643, *p* < 0.01, $\eta_{p}^{2}$ = 0.22. The interaction was non-reliable, *F* < 1.

The four right-most columns of **Table 2** indicate specific costs. Specific costs would appear as lower accuracy or longer RTs to the distractor words for studied trials compared to unstudied trials. Therefore, we compared distractor task performance during studied trials to that of unstudied trials. Furthermore, because two distractor items were presented for each category trial, we included distractor timing (simultaneous with category name onset versus delayed) as a factor in the analysis. A 2 (task: syllable, semantic) × 2 (trial: studied, unstudied) × 2 (timing: simultaneous, delayed) mixed ANOVA returned a main effect of task, indicating lower accuracy on the semantic distractor task than the syllable distractor task, *F*(1,30) = 8.18, *MSE* = 0.045, *p* = 0.008, $\eta_{p}^{2}$ = 0.21. Additionally, there was a main effect of timing, *F*(1,30) = 14.94, *MSE* = 0.013, *p* = 0.001, $\eta_{p}^{2}$ = 0.33, that was qualified by an interaction with task, *F*(1,30) = 4.86, *MSE* = 0.013, *p* = 0.04, $\eta_{p}^{2}$ = 0.14. Further analysis of the interaction suggested that, for the semantic task, accuracy was reliably reduced when distractors appeared simultaneously than when distractors were delayed (*M* = 0.59 vs. 0.71, respectively), *F*(1,15) = 21.41, *MSE* = 0.011, *p* < 0.001, but no such reduction occurred for the syllable judgment task (*M* = 0.74 vs. 0.78, respectively), *F*(1,15) = 1.21, *MSE* = 0.015, *p* = 0.29. All other effects and interactions were not reliable, all *F*s ≤ 2.06, all *p*s ≥ 0.16. There were no reliable effects or interactions associated with trial, which suggests that there were no specific costs associated with implicit memory retrieval on distractor task accuracy.

With respect to RT, the same ANOVA described in the previous paragraph returned a marginal main effect of task, indicating shorter RTs for the syllable judgment task than for the semantic task, *F*(1,30) = 3.76, *p* = 0.06, $\eta_{p}^{2}$ = 0.11. There was also a reliable main effect of timing, *F*(1,30) = 30.05, *MSE* = 170,068, *p* < 0.001, $\eta_{p}^{2}$ = 0.50, a two-way interaction between timing and trial, *F*(1,30) = 5.53, *MSE* = 13,805, *p* = 0.02, $\eta_{p}^{2}$ = 0.16, and a three-way interaction between timing, trial, and task, *F*(1,30) = 16.69, *MSE* = 13,805, *p* < 0.001, $\eta_{p}^{2}$ = 0.36. The three-way interaction suggests that specific costs occurred in the semantic task when distractors were delayed but not when distractors were presented simultaneously with category names. Specifically, RTs to delayed distractors in the semantic DA task were slower for studied trials than for unstudied trials (*M* = 1648 vs. 1478 ms, respectively, *p* = 0.04), but when the distractors were simultaneous, RTs were somewhat faster for studied trials than for unstudied trials (*M* = 1940 vs. 2037 ms, respectively, *p* = 0.08). No such pattern emerged for the syllable judgment task.

### Discussion

Divided attention at retrieval had no effect on repetition priming for the entire sample. However, for test-unaware participants, DA reduced priming when the distractor task involved semantic decisions rather than phonological decisions. We discuss this finding further in the General Discussion. The CEG task was clearly associated with global costs, indicating that the task broadly requires attentional resources. The evidence for specific costs was weak, however, emerging only for the DA-Semantic condition, only for RTs, and only for delayed distractor trials. In all other cases, no specific costs or specific benefits were observed.

The results raise several questions. First, because accuracy was lower and RTs were longer for the semantic distractor task compared to the phonological distractor task in every analysis, the effect of semantic DA on priming in test-unaware participants may reflect distractor task difficulty *per se* rather than the processing demands of the distractor tasks. We addressed this concern in Experiment 2 by examining the effects of DA on the matched explicit test of category-cued recall. If the results from Experiment 1 reflect distractor task difficulty, we would expect a similar pattern of outcomes for recall: significantly lower recall rates for the DA-Semantic condition compared to the DA-Syllable condition. Second, the baseline rate of responding with exemplars from the unstudied list in the CEG task was quite low, ranging from 0.06 to 0.08, raising the concern of floor effects reducing the magnitude of priming. We addressed that concern in Experiment 3, in which we used exemplars with a higher rate of baseline responding.

## Experiment 2

### Method

#### Participants

Forty-eight undergraduate students (32 women, 16 men), none of whom participated in Experiment 1, participated in Experiment 2 for course credit or for pay.

#### Materials, Design, and Procedure

The experiment was identical to Experiment 1 with the exception that the test was now a category-cued recall test. At test, participants were instructed to use the category names as cues to recall aloud the words that they had rated for pleasantness at the beginning of the experimental session. They were told further that not all category names would correspond to earlier-rated words, and if a category did not bring anything to mind they were to remain silent. Participants in the FA condition ignored the spoken words that accompanied the category names, whereas participants in the DA-Syllable and DA-Semantic conditions responded to the spoken words via keypress in ways appropriate to their condition while simultaneously recalling exemplars.

### Results

#### Encoding

Participants provided pleasantness ratings to 99.4, 98.5, and 99.2 percent of the words in the FA, DA-Syllable, and DA-Semantic conditions, respectively. These differences were not reliable, *F* < 1.

#### Explicit Memory

The dependent measure is the proportion of studied exemplars that were recalled minus the proportion of unstudied critical items that were falsely retrieved (unstudied critical items were recalled less than 0.5% of the time). These values were 0.42, 0.31, and 0.27 for the FA, DA-Syllable, and DA-Semantic tasks, respectively, and they were reliably different by a one-way ANOVA, *F*(2,45) = 4.57, *MSE* = 0.024, *p* = 0.02, $\eta_{p}^{2}$ = 0.17. Tukey’s HSD *post hoc* tests revealed that, relative to FA, the DA-Syllable condition was marginally lower (*p* = 0.09) and the DA-Semantic condition was reliably lower (*p* = 0.02), but, importantly, the two DA conditions did not differ from each other (*p* = 0.72).

#### Distracting Task

Global and specific costs to the distracting task were calculated in the same way as in Experiment 1, and the results are displayed in **Table 3**. Recognition produced global costs to distractor task accuracy, such that DA trials were associated with worse performance compared to baseline trials, in both DA-Syllable and DA-Semantic tasks (see **Table 3**). These impressions were confirmed in a 2 (task: syllable, semantic) × 2 (block: baseline, DA) mixed ANOVA on accuracy that returned a main effect of block, *F*(1,30) = 37.77, *MSE* = 0.013, *p* < 0.001, $\eta_{p}^{2}$ = 0.56, and a main effect of task, *F*(1,30) = 12.02, *MSE* = 0.025, *p* = 0.002, $\eta_{p}^{2}$ = 0.29. The interaction was non-reliable, *F* < 1.

**Table 3:** Experiment 2 accuracy and reaction time (RT) to distracting tasks.

	Global costs		Specific costs			
			DA-Studied		DA-Unstudied	
	Baseline	DA-Overall	Sim	Del	Sim	Del
Accuracy						
DA-Syllable	0.92	0.75	0.71	0.76	0.74	0.80
DA-Semantic	0.79	0.61	0.56	0.63	0.58	0.68
RT						
DA-Syllable	1191	1746	1967	1610	2014	1544
DA-Semantic	1334	1705	1927	1538	1904	1590

*DA, divided attention; Sim, simultaneous distractors; Del, delayed distractors*.

Recognition also produced global costs to distractor task RTs. Specifically, RTs to distractors (correct responses only) increased under DA trials than under baseline trials, and more so for syllable judgments than for semantic judgments (see **Table 3**). These impressions were confirmed in a similar ANOVA on RTs that returned a reliable effect of block, *F*(1,30) = 180.28, *MSE* = 19,051, *p* < 0.001, $\eta_{p}^{2}$ = 0.86, and an interaction between task and block, *F*(1,30) = 7.11, *MSE* = 19,051, *p* = 0.01, $\eta_{p}^{2}$ = 0.19. The main effect of task was not reliable, *F* < 1. An analysis of the interaction revealed that the RT difference between the DA-Syllable and DA-Semantic group was marginally reliable for baseline trials (*p* = 0.06), but the same difference for DA trials was not reliable (*p* = 0.50). The difference between baseline and DA trials was reliable for each group when analyzed separately, both *p*s < 0.001.

We analyzed specific costs with 2 (task: syllable, semantic) × 2 (trial: studied, unstudied) × 2 (timing: simultaneous, delayed) mixed ANOVAs, as in Experiment 1. With respect to accuracy, this analysis returned three effects, a marginally reliable main effect of trial in which distractor task accuracy was better for unstudied trials than for studied trials (*M* = 0.70 vs. 0.66, respectively), *F*(1,30) = 3.31, *MSE* = 0.01, *p* = 0.08, $\eta_{p}^{2}$ = 0.10, a reliable main effect of timing in which accuracy was better for delayed trials than for simultaneous trials (*M* = 0.72 vs. 0.65, respectively), *F*(1,30) = 23.45, *MSE* = 0.006, *p* < 0.001, $\eta_{p}^{2}$ = 0.44, and a reliable main effect of condition in which accuracy was better for the syllable judgment task than for the semantic judgment task (*M* = 0.75 vs. 0.61, respectively), *F*(1,30) = 6.15, *MSE* = 0.10, *p* = 0.02, $\eta_{p}^{2}$ = 0.17. All other effects and interactions were not reliable, all *F*s < 1. With respect to RT, the ANOVA returned only a main effect of timing in which RTs were longer for simultaneous trials compared to delayed trials (*M* = 1953 vs. 1571 ms, respectively), *F*(1,30) = 43.81, *MSE* = 106,853, *p* < 0.001, $\eta_{p}^{2}$ = 0.59. All other effects were not reliable, all *F*s ≤ 2.50, all *p*s ≥ 0.12.

### Discussion

Experiment 2 produced several notable results. First, DA at retrieval reduced explicit memory using the category-cued recall test, a result that extends previous studies of retrieval-phase DA on explicit memory to include category-cued recall (e.g., Fernandes and Moscovitch, 2000; Clarke and Butler, 2008; Lozito and Mulligan, 2010). Second, the two DA tasks had a similar impact on explicit memory, suggesting that the reduction in priming observed among test-unaware participants in Experiment 1 cannot be attributed solely to the difficulty of the semantic DA task. If that were the case, the semantic DA task would have reduced explicit memory significantly more than the syllable DA task, but that outcome did not emerge. Third, category-cued recall produced global costs to the distractor tasks, similar to that of CEG, which indicates that the category-cued recall task broadly requires attentional resources. Fourth, the pattern of specific costs on the category-cued recall task differed from that of CEG. Distractor task accuracy was reduced, albeit marginally, on studied trials compared to unstudied trials, indicating a specific cost associated with retrieving studied items. No such cost occurred when CEG was used as the test. We return to these points in the General Discussion.

## Experiment 3

Experiment 3 was a replication of Experiment 1 using a different set of stimuli for the CEG test. Recall that one concern about Experiment 1 was that baseline response rates to the CEG task were quite low, thereby raising the concern that floor effects attenuated priming. Although priming was reliable in each attention condition of Experiment 1, their magnitudes were lower than the 0.10–0.20 priming effects that are typically seen in other studies of attention and CEG priming (e.g., Schmitter-Edgecombe, 1996; Gabrieli et al., 1999; Light et al., 2000; Lozito and Mulligan, 2010). By raising baseline rates of completion, the expectation was that priming effects would better resemble those of previous studies and provide an additional test of whether retrieval-phase DA impacts priming.

### Method

#### Participants

Forty-eight undergraduate students (31 women, 17 men), none of whom participated in the previous experiments, participated in Experiment 3 for course credit or for pay.

#### Materials

We constructed a new set of 60 category exemplars from the critical, non-filler categories that were used in Experiments 1 and 2. Specifically, we relaxed the constraint that exemplars could not occur as one of the top three responses of each category in the category norms, and we chose exemplars that had a baseline probability of response that was around 0.30, similar to that of Lozito and Mulligan (2010, Experiment 3), in the category norms. This new master list was divided into two 30-item lists to serve as the studied-unstudied counterbalance, as in Experiment 1. With the exception of one category that was replaced, all category names at test (30 studied, 30 unstudied, 30 filler) were unchanged from previous experiments.

#### Design and Procedure

The design and procedure were identical to that of Experiment 1.

### Results

#### Encoding

Participants provided pleasantness ratings to 99.7, 99.4, and 99.0 percent of the critical words in the FA, DA-Syllable, and DA-Semantic conditions, respectively. These differences were not reliable, *F* < 1.

#### Implicit Memory

Proportions of critical items produced on the CEG test by the full sample are shown in the top half of **Table 4**. Baseline rates of responding were substantially higher than that of Experiment 1 and did not differ across attention conditions, *F* < 1. As expected with the higher baseline rates, priming scores were also substantially larger than those of Experiment 1. Inspection of the priming scores in **Table 4** suggests that, like Experiment 1, priming was unaffected by DA, and that impression was confirmed by a one-way ANOVA, *F*(2,45) = 0.32, *MSE* = 0.02, *p* = 0.73. Priming was reliably greater than zero when each condition was analyzed separately, all *t*(15)s ≥ 5.76, all *p*s < 0.001.

**Table 4:** Experiment 3 proportions of critical items produced on the implicit memory test of category exemplar generation (±1 SE in parentheses).

Condition	Studied items	Unstudied items	Priming
**Full sample**			
FA	0.40 (0.03)	0.23 (0.02)	0.17 (0.03)
DA-Syllable	0.44 (0.03)	0.24 (0.02)	0.21 (0.03)
DA-Semantic	0.42 (0.03)	0.23 (0.01)	0.19 (0.03)
**Unaware only**			
DA-Syllable	0.41 (0.07)	0.21 (0.05)	0.21 (0.07)
DA-Semantic	0.34 (0.05)	0.23 (0.02)	0.11 (0.04)

*FA, full attention; DA, divided attention; Unaware Only, participants who did not report awareness of the relationship between test cues and studied words*.

As in Experiment 1, participants were classified as *test-unaware*, *test-aware*, and *intentional* based on their responses to the awareness questionnaire. The lower half of **Table 4** presents the proportions for the six DA-Syllable and five DA-Semantic participants that were classified as test-unaware (no FA participant was classified as test-unaware). Although priming in the DA-Semantic condition was about half that of the DA-Syllable condition, the difference did not achieve significance, *t*(9) = 1.10, *p* = 0.30, most likely owing to the low statistical power in this analysis.

#### Distracting Tasks

Global and specific costs were calculated in the same manner as described in Experiment 1, and the results are displayed in **Table 5**. Global costs occurred for both accuracy and RT measures, and were generally similar to those seen in Experiment 1. A 2 (task: syllable, semantic) × 2 (block: baseline, DA) mixed ANOVA on accuracy indicated two main effects, such that accuracy was lower on the DA block compared to baseline, *F*(1,30) = 142.21, *MSE* = 0.005, *p* < 0.001, $\eta_{p}^{2}$ = 0.83, and accuracy was lower on the semantic task than for the syllable judgment task, *F*(1,30) = 8.62, *MSE* = 0.014, *p* < 0.01, $\eta_{p}^{2}$ = 0.22. The interaction was non-reliable, *F* < 1. The same ANOVA applied to RT revealed only a main effect of block, such that RTs increased for the DA block relative to baseline, *F*(1,30) = 212.30, *MSE* = 21,924, *p* < 0.001, $\eta_{p}^{2}$ = 0.88. All other *F*s < 1.

**Table 5:** Experiment 3 accuracy and reaction time (RT) to distracting tasks.

	Global costs			Specific costs		
			DA-Studied		DA-Unstudied	
	Baseline	DA-Overall	Sim	Del	Sim	Del
Accuracy						
DA-Syllable	0.95	0.76	0.73	0.78	0.70	0.82
DA-Semantic	0.88	0.66	0.62	0.72	0.61	0.68
RT						
DA-Syllable	1145	1720	2056	1491	2067	1489
DA-Semantic	1229	1732	2030	1488	2006	1456

*DA, divided attention; Sim, simultaneous distractors; Del, delayed distractors*.

Specific costs on accuracy were analyzed with a 2 (task: syllable, semantic) × 2 (trial: studied, unstudied) × 2 (timing: simultaneous, delayed) mixed ANOVA. Two main effects were obtained in this analysis. The effect of timing indicated that responses to distractor trials were less accurate if distractors were presented simultaneously with the category cue compared to delayed distractors, *F*(1,30) = 22.51, *MSE* = 0.01, *p* < 0.001, $\eta_{p}^{2}$ = 0.43. The effect of task indicated that responses to the semantic task were less accurate overall compared to the syllable task, *F*(1,30) = 5.40, *MSE* = 0.06, *p* = 0.03, $\eta_{p}^{2}$ = 0.15. All other *F*s ≤ 1.61, all *p*s ≥ 0.21. The same ANOVA applied to RT revealed only a main effect of timing, such that RTs were longer for simultaneous trials compared to delayed trials, *F*(1,30) = 59.19, *MSE* = 168,903, *p* < 0.001, $\eta_{p}^{2}$ = 0.66. All other *F*s < 1. Note that trial type did not enter into any reliable effects or interactions, suggesting that there were no specific costs associated with implicit memory retrieval.

## General Discussion

The overarching goal of the present study was to determine whether conceptual implicit memory was sensitive to DA at retrieval. Previous studies have reported that priming in a variety of implicit memory tasks is insensitive to retrieval-phase DA, a result that supports the automatic retrieval view (Clarke and Butler, 2008; Lozito and Mulligan, 2010). However, previous studies did not vary the similarity of processing required by the distracting task and the implicit memory task. The present study did so using the conceptually driven implicit memory task of CEG and using distracting tasks that either required judgments about the sound of words or the meaning of words. The former condition (DA-Syllable) is assumed to involve phonological processes in the distracting task that differ from the semantic or conceptual processes required by the implicit memory task, and the latter condition (DA-Semantic) is assumed to involve similar semantic processes across the two tasks. Process-specific DA effects can occur in explicit memory (Fernandes and Guild, 2009; Wammes and Fernandes, 2015) but to our knowledge this study is the first to examine whether implicit memory is susceptible to the same sort of effect. In this discussion, we first consider the impact of our DA tasks on implicit and explicit memory, then we turn our attention to the impact of the CEG and category-cued recall tasks on general and specific costs to distractor task performance.

The first major result is that conceptual implicit memory was unaffected by any type of retrieval-phase DA task when full samples were considered (Experiments 1 and 3). We find this outcome surprising, considering the fact that we created an especially powerful set of conditions in which a DA effect on implicit memory could be seen. Specifically, we used the CEG test, a conceptually driven implicit memory task that requires stimulus production. Both TAP and the identification-production framework agree that attention is necessary for full priming on that task, and encoding-phase DA effects on CEG priming are robust (Spataro et al., 2011). Thus, among implicit memory tasks, the CEG task should be most likely to reveal any DA effects in priming that may exist. We also used distracting tasks that required frequent rather than occasional responding and paid careful attention to the timing of distracting and implicit memory test stimuli. Response frequency and timing of tasks are both factors that are known to modulate DA effects on priming in some implicit memory tasks (Mulligan, 2003; Mulligan et al., 2007). Despite those design features, there was no hint of a DA effect across two experiments, at least when the full samples in both experiments were considered. Note that, if anything, the DA effect on priming was in the opposite direction, that is, numerically greater priming under DA conditions relative to FA. That fact indicates that the failure to find statistically reliable differences among conditions is not likely to reflect trends toward a DA effect on priming that were undetected due to a lack of power.

The priming outcomes for the full samples in Experiments 1 and 3 join other studies that have found support for the automatic retrieval hypothesis (Clarke and Butler, 2008; Lozito and Mulligan, 2010). According to that hypothesis, implicit memory is unaffected by retrieval-phase division of attention. The novel contribution of the present study is that the automatic retrieval hypothesis applies to implicit memory regardless of the similarity in processes between the distracting task and memory task. That is, conceptual priming appears to be insensitive to material-specific interference as well as process-specific interference by retrieval-phase DA. The results are less well understood by the TAP and identification-production perspectives, however. According to TAP (Roediger and McDermott, 1993), conceptual processes are affected by DA and should therefore impact performance on conceptually driven implicit memory tasks, of which the CEG task is a type. According to the identification-production account (Gabrieli et al., 1999), attention is critical for production priming tasks, and the CEG task is unambiguously such a task. The data therefore favor the automatic retrieval view.

Participants classified as unaware in Experiments 1 and 3 showed priming effects that were reduced by DA. In Experiment 1, priming in the DA-Semantic task was about half that of the DA-Syllable and FA conditions, and in Experiment 3, priming in the DA-Semantic task was numerically lower than that of the DA-Syllable condition. While these results seem to challenge the automatic retrieval view and suggest the existence of process-specific interference, we exercise caution in interpreting these outcomes. First, the DA-related reductions in priming were not general effects, but emerged only among unaware participants. Second, the findings are based on very small subsamples of the original FA and DA groups. Third, evidence for process-specific interference would be obtained if priming was reliably reduced in the DA-Semantic condition compared to the DA-Syllable condition, but that reduction was marginal in Experiment 1 and non-reliable in Experiment 2, and a combined analysis of all unaware participants in Experiments 1 and 3 did not reveal reliable differences among conditions (*p* = 0.09). Finally, the analyses of specific costs on the distractor tasks do not provide additional evidence against the automatic retrieval view (a specific cost in the DA-Syllable condition was found for RT in Experiment 1, but no specific costs were observed in Experiment 3). Nevertheless, the results from the unaware participants hint at the possibility that process-specific interference in implicit memory could occur under very specific conditions (conceptually driven tests, unaware participants). Future studies with larger numbers of unaware participants may find results that challenge the automatic retrieval view, however, we believe the weight of evidence currently favors the automatic retrieval view.

The effects of DA on explicit memory differed from those seen on implicit memory. In explicit memory, both semantic and phonological distracting tasks worked to reduce performance on category-cued recall with no reliable difference between the two DA conditions (Experiment 2). This outcome fits well with the view that retrieval-phase DA can negatively affect explicit memory under some situations, particularly when the distracting task and memory task employ similar materials (Fernandes and Moscovitch, 2000). However, process-specific interference in explicit memory was not observed. Process-specific interference has been most commonly observed using non-verbal stimuli such as faces or visuospatial patterns; such interference effects have been examined with words less often but appear to be less commonly observed. In one study (Fernandes and Guild, 2009), retrieval-phase DA effects on recognition for words were numerically lower when the distracting task involved phonological judgments (does the sound of a consonant letter, presented visually, rhyme with “ee”?) rather than visuospatial judgments (does a consonant letter contain a curved line?), however, this difference was not reliable in a direct comparison. Other studies that have used semantic and phonological distracting tasks similar to those described in the present investigation have also shown little difference in recall performance between DA conditions (Fernandes and Moscovitch, 2000).

Turning to the distractor task, we found that the automatic retrieval hypothesis gained support through the analysis of global and specific costs. As expected, global costs emerged in the syllable and semantic judgment tasks when performed under DA conditions in the presence of the CEG task. Global costs, however, cannot say whether implicit memory retrieval is automatic, only that the CEG task *per se* requires attentional resources. Only an analysis of specific costs provides a focused test of the automatic retrieval hypothesis, and that analysis generally supports that hypothesis. The automatic retrieval hypothesis would be refuted if performance decrements occurred on the distracting task for studied trials compared to unstudied trials. As mentioned earlier, this outcome occurred in only one situation in Experiment 1 (RTs for delayed distractors in the DA-Semantic condition) and was not replicated in Experiment 3. Most analyses revealed no difference between studied and unstudied trials, or uncovered specific benefits in which distracting performance was facilitated during studied trials. The facilitation may be due to the relative availability of resources when studied material is more fluently and quickly processed at test (see Lozito and Mulligan, 2010, for further discussion).

A somewhat different pattern of secondary task costs emerged for category-cued recall. As expected, global costs occurred on distractor task performance and may reflect the attention-demanding costs of establishing retrieval mode (see, e.g., Naveh-Benjamin et al., 1998). A trend toward specific costs that was observed in distractor task accuracy may highlight the use of a metacognitive strategy, such that participants spend more time or make more effort to retrieve words from category cues believed to be helpful. Alternatively, they may terminate the retrieval process earlier for category cues that are judged to be less helpful. Either or both patterns would create more resources available for unstudied trials, and thus better performance on distracting tasks, together with fewer resources available for studied trials, and thus worse performance on distracting tasks (Lozito and Mulligan, 2010).

In summary, the present experiments investigated retrieval-phase DA on implicit memory and reinforce the conclusions from previous studies that implicit memory retrieval is automatic. On the whole, implicit memory is unaffected by retrieval-phase DA regardless of whether distracting tasks use similar materials as the memory task, and now regardless of whether distracting tasks use similar processes as the memory task. It remains to be seen in further research whether (a) process-specific interference effects in implicit memory emerge with larger samples of unaware participants, and (b) priming in other implicit memory tests, such as word-stem completion, lexical decision, and semantic verification, is also automatic when distractor tasks overlap with the retrieval processes used in those tests.

## Conflict of Interest Statement

The authors declare that the research was conducted in the absence of any commercial or financial relationships that could be construed as a potential conflict of interest.

## Footnotes

1 Two studies have also examined retrieval-phase DA effects on word-stem associative priming in which context-target word pairs are studied (e.g., *hat – limes, gutter – coat, dove – sting*) and participants respond to test items consisting of target word stems presented with the same context word that appeared at encoding (*hat – lim___*) or with a different context word (*gutter – sti____*). Associative priming occurs when targets are produced more often in the same context than in a different context. Gooding et al. (1999) found no DA effect on associative priming when mental arithmetic was the distracting task at retrieval, however, Kinoshita (1999) found a DA effect on associative priming when a digit monitoring task was the distracting task. The present study focuses on priming of single items rather than priming of new associations, and thus these mixed results are not considered further.
